# Caregivers’ Assessment of the Sensory Processing Patterns Exhibited by Children with Autism in the Gulf Region

**DOI:** 10.1007/s10803-023-05937-4

**Published:** 2023-05-12

**Authors:** Rehab H. Alsaedi, Suzanne Carrington, James J. Watters

**Affiliations:** 1 Faculty of Creative Industries, Education and Social Justice, Queensland University of Technology (QUT), Victoria Park Road, Kelvin Grove, Brisbane, QLD 4059 Australia; 2 The Department of Special Education, Taibah University, Janadah Bin Umayyah Road, Madinah, 41477 Saudi Arabia

**Keywords:** Sensory processing, Autism spectrum disorder, Gulf region, Child Sensory Profile-2, Hyposensitivity, Hypersensitivity

## Abstract

This study explored the nature, prevalence, and developmental profiles of sensory processing disorders among children with autism spectrum disorder (ASD). The participants comprised 119 children with ASD and 30 typically developing children and their parents. The Child Sensory Profile-2 was used to assess the children’s sensory processing characteristics. The children with ASD exhibited elevated sensory processing difficulties. Deficits were observed in all the sensory modalities among the children with ASD, except the visual processing modality. Age-related improvements were observed in most sensory processing domains, although non-significant differences were noted in three domains. These findings should enhance understanding of the sensory challenges faced by children with ASD and contribute to the development of individually tailored, targeted, and age-specific therapeutic interventions.

Sensory processing disorders (SPDs) are disturbances in the “functions related to sensation occurring in the central nervous system,” including “reception, modulation, integration, and organization of sensory stimuli gathered from the seven sensory systems” (Bundy et al., 2002, p.480; Walbam, 2014). Significant sensory processing abnormalities can co-occur with clinical disorders seen in childhood, including autism spectrum disorder (ASD). Indeed, prior studies involving standardized parent-rated questionnaires have estimated that 45–95% of individuals with ASD demonstrate some degree of sensory disturbance (Green et al., 2016; Leekam et al., 2007; Tomchek & Dunn, 2007). Therefore, SPDs, particularly sensory over- and under-responsivity, are now included in the diagnostic criteria for ASD (American Psychiatric Association [APA], 2013a), which has led to renewed research interest in sensory processing among the ASD population.

Sensory abnormalities can compound the neurological deficits experienced by individuals with ASD, resulting in problematic behaviors, poor adaptation in daily life, and limited participation in productive activities (Suarez, 2012; Yasuda et al., 2016). Furthermore, evidence suggests that SPDs may contribute to the exacerbation of the clinical presentation of ASD (Adamson et al., 2006; Kern et al., 2007; Zachor & Ben-Itzchak, 2013). The negative effects of SPDs can extend to the daily routines of children with ASD, which can cause elevated parental stress levels (Ben-Sasson et al., 2013). Given the knock‑on effects of SPDs with regard to a child’s developing brain and behavior, it is important that therapists consider an SPD diagnosis and appropriate treatment early in a child’s life.

There has been some debate among researchers and practitioners as to whether SPDs represent a specific feature of ASD. Prior studies have reported that unusual sensory symptoms are well established across all ages and severity levels in individuals with ASD (Ben-Sasson et al., 2009, 2019; Ermer & Dunn, 1998; Joosten & Bundy, 2010; Suarez, 2012). Evidence has also indicated that a distinct pattern of sensory abnormalities differentiates individuals with ASD from individuals with other clinical diagnoses (Joosten & Bundy, 2010). By contrast, other studies have found that sensory characteristics are neither unique nor specific to ASD, having been described in individuals with various developmental disorders (Cheung & Siu, 2009; Rogers & Ozonoff, 2005; Rogers et al., 2003). Due to this inconsistency, some researchers have strongly questioned the usefulness of including the sensory item within the diagnostic criteria for ASD (Grapel et al., 2015; Volkmar & Reichow, 2013), although sensory symptoms remain important intervention targets in those with ASD.

Prior studies have sought to identify patterns of sensory dysfunction within the ASD population, which has included the identification of atypical responses manifesting in alternating states of excitement and inhibition that may be attributed to an imbalance or poor processing of sensory input in the brain due to the individual’s neurological threshold being either “high” or “low” (Dunn, 2014). According to Dunn’s (1997, 2014) sensory processing framework, there are four patterns of sensory processing dysfunction: registration (high threshold and passive self-regulation), seeking (high threshold and active self-regulation), sensitivity (low threshold and passive self-regulation), and avoiding (low threshold and active self-regulation). When individuals exist at the extreme edges of these patterns and so exhibit either hypersensitivity or hyposensitivity, which impacts their occupational performance, meaningful participation, and general well-being, they are said to have an SPD (Dunn, 2014). Here, the avoiding and sensitivity quadrants are highly correlated and designed to indicate sensory over-responsivity or a low threshold (Ben-Sasson et al., 2008; Miguel et al., 2017).

By contrast, the registration quadrant is designed to capture those sensory processing behaviors that indicate under-responsivity or a high threshold (Kern et al., 2006). Sensory seeking is another sensory pattern thought to be seen in children with ASD due to a state of low arousal (Siri & Lyons, 2014). In this state, the neurons tend to have a much higher threshold for activation. Yet, the findings of Liss et al. (2006) suggested sensory seeking to be a compensatory response to over-arousal.

While many studies have identified sensory seeking behavior to be a distinct third domain of sensory differences (Baranek et al., 2006; Lane et al., 2011; Rogers & Ozonoff, 2005), other research in the ASD field has categorized sensory seeking behavior as an aspect of hyposensitivity (Dunn, 2007). Some researchers have indicated that the sensory seeking pattern remains unclear, possibly in part due to the varying conceptualizations of the associated symptoms (Schauder & Bennetto, 2016). In the present study, the sensory seeking domain was considered an aspect of hyposensitivity. Nevertheless, it is important to recognize that the sensory scores represent a combination of the scores for the seeking, avoiding, registration, and sensitivity items. Moreover, it must be remembered that almost everyone engages in each quadrant, as they involve separate but related concepts.

Some studies have suggested hyporesponsiveness (registration) to be the most common pattern of atypical sensory-related behavioral problems in children with ASD (Baranek et al., 2006; Ben-Sasson et al., 2007; Rogers & Ozonoff, 2005; Schoen et al., 2009), while others have indicated hypersensitivity to be the most commonly impaired response (Ben-Sasson et al., 2019; Nieto et al., 2017; O’Brien et al., 2009). Moreover, a review study highlighted how sensory symptoms are seen in relation to several sensory modalities among those with ASD, although the auditory and touch modalities are most commonly affected (Ashburner et al., 2008; Fernández-Andrés et al., 2015; Sanz-Cervera et al., 2017; Tomchek & Dunn, 2007; Wiggins et al., 2009). However, the evidence remains inconclusive, meaning that children with ASD may fall within one or more of the classifications (DeBoth & Reynolds, 2017).

Determining whether chronological age impacts SPDs in the ASD population represents an important issue, as the maturational processes associated with the various sensory processing characteristics may strongly influence the behavioral manifestation of ASD symptoms (Baranek et al., 2019). Unfortunately, the available results are inconsistent with regard to the relationship between sensory symptoms and chronological age. Some studies have identified a difference between the sensory profiles of children and adults with ASD, highlighting how SPDs appear to peak during childhood and then lessen with age (Baranek et al., 2006, 2007; Caminha & Lampreia, 2012; Goldstein et al., 2006; Henshall, 2008; Kern et al., 2006, 2007; Leekam et al., 2007). Other studies have suggested that SPDs remain stable throughout an individual’s lifetime (Billstedt et al., 2007; Crane et al., 2009). Furthermore, some researchers have found that the relationship between sensory symptoms and age might vary according to the sensory domain in question (Little et al., 2018). These inconclusive results regarding the developmental nature of atypical sensory processing in children with ASD might stem from differences in the methodologies, comparison groups, age groups, analytical procedures, and other aspects of the various research protocols in this field (Baranek et al., 2013).

Sensory abnormalities have been widely reported within the ASD literature in the Western cultural context; however, there is a lack of evidence concerning SPDs in non-Western cultures, including the Arabic Gulf culture (Al-Heizan et al., 2015). Although individuals with neurological disorders might be expected to show similar symptoms across different cultures, there is a growing body evidence that sensory characteristics may be influenced by culture (Al-Heizan et al., 2015; Caron et al., 2012). Thus, it is important to assess sensory processing in children from every culture (Al-Heizan et al., 2015), as such research may provide a useful foundation for the design and adaptation of culturally sensitive assessment tools.

This study explored the distribution of SPDs among a large sample of children with ASD from the Gulf region using the Child Sensory Profile-2 (CSP-2). Based on prior findings, it was hypothesized that children with ASD would receive scores outside the normal range for at least one sensory processing domain. The clinical levels of the identified sensory abnormalities were determined based on the criterion referenced in the CSP-2 classification system (Chen et al., 2009; Simpson et al., 2019), although no clear prediction could be made with regard to any individual sensory modality, behavioral construct, or sensory response pattern within the sensory domains most commonly impaired in children with ASD. In addition, this study compared the sensory processing profiles of children with ASD with those of their typically developing (TD) peers. This was intended to facilitate a more in-depth understanding of the daily struggles that such difficulties entail for children with ASD and to provide valuable insights that should assist in the design and implementation of more appropriately tailored interventions. It was hypothesized that children with ASD would present more sensory abnormalities than TD children across all the CSP-2 domains. Moreover, this study applied a cross‐sectional design to investigate age-related differences in the sensory processing profiles of children with ASD. Given the divergent findings concerning this issue, the nature of any age-related differences in the sensory processing characteristics of individuals with ASD remains unclear. As such, this study formed no specific hypothesis and instead conducted a thorough exploration of the issue.

## Methods

### Study Participants

The participants in this study were involved in a larger study on neurobehavioral problems in children with ASD. More specifically, the children in the ASD and TD groups (aged 6–12 years) and their parents also participated in this study on a voluntary basis. Their data were collected using the CSP-2 (Dunn, 2014). The participants were recruited from three Gulf states: Bahrain, Saudi Arabia, and the United Arab Emirates (UAE).

The initial ASD sample comprised 180 participants. Thirteen participants with ASD (7.22%) were excluded due to their intelligence quotient (IQ) scores being unavailable, while 11 participants (6.11%) were excluded because they failed to reach the suggested cut-off for ASD on the two screening measures used to confirm the clinical diagnosis of ASD. Another 12 participants (6.67%) were excluded because their parents did not want to commit to further participation in the research, while 24 participants (13.33%) were excluded because they were unable to complete the neurological laboratory tasks involved in the larger study. One participant (0.56%) was excluded due to taking amphetamine medication. These exclusions led to the ASD group comprising 119 participants (95 males and 24 females) who were recruited from three types of educational institutions: a full-inclusion school setting, a partial-inclusion school setting, and a specialized autism school or center.

All the children with ASD had been clinically diagnosed by experienced pediatricians or neurologists based on the criteria within the Diagnostic and Statistical Manual of Mental Disorders, Fourth Edition, Text Revision (DSM-IV-TR) and the Childhood Autism Rating Scale (CARS). For the purposes of diagnostic confirmation and symptom severity determination, all these participants were assessed using the Gilliam Autism Rating Scale, Third Edition (GARS-3; Gilliam, 2013) and the Michigan Autism Spectrum Questionnaire (MASQ; Ghaziuddin & Welch, 2013). In most cases, these two instruments were completed by one parent. Furthermore, the Clinician-Rated Severity of Autism Spectrum and Social Communication Disorders (CRSASSC; American Psychiatric Association [APA], 2013b) measure was used to independently assess the severity of the participants’ ASD symptoms.

The inclusion criteria for the ASD group were as follows: (i) a score of ≥ 71 on the GARS-3, indicating a diagnosis of ASD to be very likely; (ii) an IQ score of ≥ 70, which ensured that any identified performance differences were not attributable to lower cognitive ability; (iii) a score of ≥ 22 on the MASQ, which is the optimal cut-off point for high-functioning ASD cases; and (iv) ASD symptoms categorized as ≥ level-one severity (requiring mild support) based on the CRSASSC. The exclusion criteria were as follows: (i) the presence of comorbid ASD-related medical conditions or other neurological conditions; and (ii) hearing and/or visual acuity not corrected to within normal limits. The exclusion criteria were verified by interviewing the parents and reviewing the children’s medical records.

In terms of the TD group, 105 prospective participants and their parents were initially invited to participate. Of those 105, the parents of 55 (52.4%) consented to participate in the research, while the parents of 50 (47.6%) declined to participate. The initial TD sample, therefore, comprised 55 participants. Six participants (10.91%) with learning difficulties were excluded from the study, while nine participants (16.36%) later withdrew their consent to participate. Another ten participants (18.18%) were excluded due to incomplete assessments. This resulted in a total of 30 TD participants being included in the study (24 males and 6 females). The TD participants were all recruited from mainstream primary schools. To be included in the analyses, the TD participants had to be free of any neurological or psychiatric disorders, psychotropic medication, or family history of ASD.

Given the unequal sample sizes of the two study groups, group-wise matching was used rather than pair-wise matching. As a result, the participants were group-wise matched based on their chronological age, gender, handedness (right or left, as assessed using a standard questionnaire; Oldfield, 1971), and non-verbal IQ (as measured using Raven’s colored progressive matrices [RCPM]; Raven et al., 1998), which was used only for matching between the study groups and parental education levels. The group comparisons of the variables were not statistically significant (all p > 0.05). The participants’ characteristics are shown in Table 1.

**Table 1:** Descriptive data for the group comparisons

Comparison Criteria	Target Group (N = 119)		Comparison Group (N = 30)					
	Children with Autism		Typically Developing Children					
Characteristic	Mean or N	SD or %	Mean or N	SD or %	t/χ 2	p	d	Adequacy
Age (M/S)	8.72	1.96	9.06	1.42	1.05	0.71	0.21	Matched
Gender (% M/F)	M = 95/F = 24	79.8/20.2	M = 24/F = 6	80.0/20.0	0.00	0.98	-	Matched
Handedness (% R/L)	R = 104/L = 15	87.4/12.6	R = 23/L = 7	76.7/23.3	2.191	0.14	-	Matched
Non-Verbal IQ (M/S)^a^	29.76	1.92	29.80	2.64	0.69	0.06	0.17	Matched
Father’s Education (% Secondary/College Degree)	67/52	56.3/43.7	16/14	53.3/46.7	0.09	0.77	-	Matched
Mother’s Education (% Secondary/College Degree)	78/41	65.5/34.5	20/10	66.7/33.3	0.01	0.91	-	Matched

*SD* standard deviation; n (% of total)

*Continuous data were analyzed by means of a t-test, whereas categorical variables were analyzed using a χ2 test

^a^The possible raw scores on the test ranged from 0 to 36

### Screening Questionnaires

It is vital to use standard, reliable, and valid tools when assessing the diagnosis and symptoms of ASD (Scharoun et al., 2014). The following screening measures were used to assess the severity of the participants’ ASD symptoms.

#### GARS-3

The researcher had previously translated the GARS-3 into Arabic and made certain cultural adaptations to facilitate its use in the Gulf region after obtaining written permission from the publisher (Pro-Ed). The GARS-3 is designed to help teachers, parents, and clinicians to identify ASD and evaluate its severity. It comprises 56 items based on the diagnostic criteria listed in the DSM-V, which describe the characteristic behaviors of individuals with ASD. The GARS-3 is categorized into six subscales: restrictive, repetitive behaviors, social interaction, social communication, emotional responses, cognitive style, and maladaptive speech. Mute individuals are assessed using the four-subscale form rather than the six-subscale form. As a measure of ASD, the GARS-3 has proven to have high validity and reliability (Gilliam, 2013). In the present study, the internal reliability of the total GARS-3 scale was 0.95 for Autism Index 6 and 0.93 for Autism Index 4. Both figures indicate the high reliability of the measure. None of the subscales had a Cronbach’s alpha lower than 0.70.

#### MASQ

The MASQ rating scale comprises ten items used to identify those with high-functioning ASD. Respondents answer each item on a four-point scale ranging from 0 (“Not at all”) to 4 (“Very much”), yielding a maximum total score of 30. According to the MASQ, the optimal cut-off score is 22 when identifying those who were formerly (i.e., before the most recent update to the DSM-V) said to have Asperger’s syndrome or high-functioning autism. The Cronbach’s alpha coefficient calculated for the internal consistency of the total MASQ score among the present sample was found to be adequate (0.81).

#### CRSASSC

The CRSASSC scale is used to assess individuals’ symptom severity and support needs in two areas: (i) social communication and (ii) restricted and repetitive behaviors. There are three possible support levels: level one (“requiring support”), level two (“requiring substantial support”), and level three (“requiring very substantial support”) (APA 2013). The identified support level can help in understanding whether a person is “high functioning” or more significantly impaired. The severity levels for each item are reported separately, meaning that an overall severity score should not be calculated. The CRSASSC scale has demonstrated appropriate interobserver reliability (Ellison et al., 2019). However, little research is currently available regarding the utility of the severity scale, although the associated rating scores seem likely to exhibit some degree of validity. The scores tend to be correlated with one standardized measure of the severity of ASD as well as with other clinical features of the disorder, particularly cognitive and behavioral functioning (Mazurek et al., 2019). Yet, the DSM-V’s severity scale arguably does not exhibit sufficient validity to justify its use as the sole means of assessing the severity of ASD/classifying children with ASD according to the severity of their symptoms (Ellison et al., 2019).

### Assessment Measure

#### CSP-2

The CSP-2 is an adapted version of a widely used sensory processing measure (Dunn, 1999) intended to evaluate children’s sensory processing patterns in the context of everyday life (Little et al., 2017; Miguel et al., 2017). It is comprised of four quadrants: sensation avoiding, sensory sensitivity, sensation seeking, and registration. There are six sensory systems (auditory, visual, touch, movement, body position, and oral) and three behavioral sections (conduct, social/emotional, and attention). The caregiver questionnaire contains 86 items, and parents are asked to rate their child’s responses to everyday events on a five-point Likert-type scale (5 = almost always, 4 = frequently, 3 = half the time, 2 = occasionally, or 1 = almost never), with higher and lower scores indicating greater differences. The CSP-2 also includes a “0 = does not apply” option for use when necessary. There are cut-off scores for each summary raw score, which are based on a bell curve. Scores one standard deviation or more from the mean are expressed as “more than others” or “less than others,” as appropriate. Scores two standard deviations or more from the mean are expressed as “much more than others” or “much less than others,” as appropriate. The test takes around 20 min to administer.

The CSP-2 was normed using a large national sample (n = 697), which demonstrated its strong psychometric properties. In fact, its internal consistency alpha values ranged from 0.60 to 0.90, its test–retest reliability values ranged from 0.87 to 0.97, and its interrater reliability values ranged from 0.92 to 0.99 (Dunn, 2014). In terms of the four quadrants, Simpson et al. (2019) reported the Cronbach’s alphas for the seeking, avoiding, sensitivity, and registration quadrants to be 0.69, 0.83, 0.75, and 0.75, respectively. The content validity, criterion-related validity, and construct validity are also detailed in the manual (Dunn, 2014).

In the present study, each of the sensory processing patterns, sensory systems, and behavioral sections showed adequate levels of internal consistent, ranging from 0.73 to 0.95 for the ASD sample and 0.71–0.90 for the TD sample, which is considered adequate for research purposes. Thus, these internal consistency levels provide evidence of the adequate reliability of the scale.

### Procedures

This study was conducted as part of a larger investigation approved by Queensland University of Technology’s Human Research Ethics Committee. Children aged 6–12 years were recruited from autism centers and schools that voluntarily agreed to participate in the study. The parents of the selected children provided written informed consent to participate in the research.

As part of the screening process, the parents were asked to provide information about the severity of their child’s ASD by completing the GARS-3 and MASQ. In addition, the children with ASD underwent an assessment using the CRSASSC scale, which was conducted by the researcher to determine the severity of their ASD. The parents were also asked to complete part of the Edinburgh Handedness Inventory (Oldfield, 1971) to determine the participants’ hand dominance (left- or right-handed). Moreover, the RCPM were administered to provide an estimation of the participants’ intellectual ability. The parents of the TD children were asked to complete the demographic questionnaire and handedness questionnaire. The intellectual ability of the TD children was measured using the RCPM. The researcher verified that the children in both groups met the inclusion criteria based on parental reports and a school record review. Finally, the parents of all the participants were asked to complete the CSP-2.

### Statistical Analysis

All data analyses were performed using the Statistical Package for the Social Sciences (SPSS) version 23.0. In terms of the CSP-2, the cut-off criterion for each individual subscale was used to estimate the proportions of the elevated and reduced scores relative to normative performance expectations. Descriptive statistics (mean, standard deviation, range, minimum, and maximum) were calculated for the raw scores for the key CSP-2 variables. The independent samples t-test was used to determine the extent of the variation in performance between the groups in relation to the study variables. Due to the large number of comparisons performed, the Bonferroni correction was used to avoid increasing the risk of a type I error occurring (Shasha & Wilson, 2011). The effect sizes were calculated using Cohen’s d, where d = 0.2 is considered small, d = 0.5 is considered medium, and d = 0.8 is considered large.

To explore the effect of age on performance, a regression analysis was performed with the child’s age as the predictor variable and the raw score of the child’s performance on each subscale as the outcome of interest. As there were several predictor variables included in the regression analysis, the level of statistical significance was set as p < 0.01 to avoid type I errors. Furthermore, to avoid type II errors, items with p values of < 0.05 were also noted. The magnitude of the R-squared (R2) value was interpretated according to Cohen’s (1988) guidelines, with the values of Cohen’s ƒ2 = 0.02, 0.13, and 0.26 being considered to indicate a small, medium, and large effect size, respectively.

## Results

First, the findings regarding the estimated distribution rates of the sensory processing disorders among the children with ASD will be presented. The ASD group’s scores for each CSP-2 domain were compared with the criterion referenced in the instrument’s assessment manual, which is based on a bell-curve normal distribution (Dunn, 2014). Next, the results of the comparison between the children with ASD and their TD peers, as performed using the Arabic version of the CSP-2, will be discussed. Finally, the identified age-related changes in the sensory symptoms observed in the children with ASD will be examined.

### Distribution of Scores for the Sensory Domains and Prevalence of Sensory Dysfunctions Among the Children with ASD

Table 2 presents descriptive statistics concerning the variables investigated in this study, including the mean of the total raw scores, standard deviations, and slight negative skewness, as well as the ASD group’s classifications according to the four sensory processing patterns, six sensory systems, and three behavioral groupings associated with sensory processing.

**Table 2:** Descriptive Statistics Concerning the ASD Participants’ Sensory Profile Raw Scores Across the CSP-2 Domains

Subscale	ASD Sample (N = 119)							*Classification*
	RST	M	SD	Range	SE	Lower	Upper	
Sensory Quadrants								
Seeking (20–47)	/95	55.87	12.18	30–84	1.12	53.66	58.09	More than others
Avoiding (21–46)	/100	59.39	8.31	41–80	0.76	57.89	60.90	Much more than others
Sensitivity (18–42)	/95	51.44	8.74	33–69	0.82	53.38	56.64	More than others
Registration (19–43)	/110	61.51	14.03	33–91	1.29	58.97	64.06	Much more than others
Sensory Section								
Auditory (10–24)	/40	26.19	5.133	12–36	0.47	25.26	27.13	More than others
Visual (9–17)	/30	15.23	4.02	6–23	0.37	14.50	15.96	Like the majority of others
Touch (8–21)	/55	30.26	7.74	14- 46	0.71	28.86	31.67	Much more than others
Movement (7–18)	/40	22.66	6.82	8–38	0.63	21.42	23.89	More than others
Body Position (5–15)	/40	19.84	6.33	8–32	0.58	18.69	20.99	Much more than others
Oral (8–24)	/50	26.71	7.69	12–43	0.70	25.32	28.11	More than others
Behavioural Section								
Conduct (9–22)	/45	27.28	6.66	14–44	0.61	26.07	28.49	More than others
Social/Emotional (13–31)	/70	42.62	6.49	27–57	0.59	41.44	43.80	Much more than others
Attentional (9–24)	/50	31.42	5.54	13–45	0.51	30.41	32.43	Much more than others

The figures given in brackets correspond to the normal expected range. Lower scores indicate less or much less sensory functioning than that exhibited by individuals in the normal range, while higher scores indicate more or much more sensory functioning than that exhibited by individuals in the normal range. *RST* raw score total, *ASD* autism spectrum disorder

As shown in Table 2, based on the normative data, the participants in the ASD group recorded elevated scores across all the CSP-2 domains, except for the visual sensory processing items, which indicates the presence of more sensory symptoms than seen in individuals within the normal range. The mean score for each subscale was beyond the normal cut-off range, with the most common responses being “more than others” and “much more than others.” This pattern was replicated in relation to 12 of the 13 CSP-2 summary scores.

### Differences Across the Sensory Quadrant Scores

In terms of the four sensory quadrants, the children with ASD scored higher than the normative age expectations with regard to the seeking quadrant, which indicates that they may actively seek increased sensory input. For the avoiding quadrant, the children with ASD were classified within the range of “much more” than normative age expectations, which suggests hyper- or over-sensitive children who may move away from sensory input at a higher rate than other children. As for the sensitivity and registration quadrants, the scores of the children with ASD met the criteria for the “more than others” and “much more than others” classifications, respectively. The children with ASD had much higher scores when compared with the normal range. Those children who scored highly in the sensitivity quadrant tended to notice sensory inputs at a higher rate than others, indicating them to be hyper- or over-sensitive, while the children who scored highly in the registration quadrant tended to miss available sensory inputs at a higher rate than others, indicating them to be hypo- or under-sensitive.

**Fig. 1:**
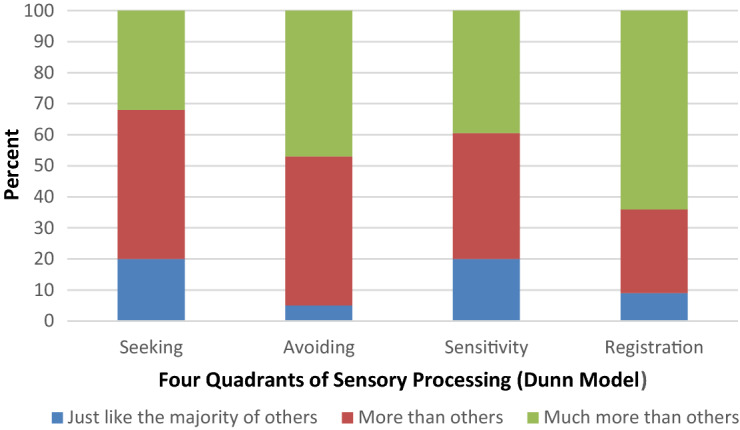
Sensory profile scores among the 119 children with ASD across the sensory quadrant scores

As can be seen in Fig. 1, 63.9% of the sample were classified within the “much more than others” or “MM2” range for the registration quadrant, 47.1% for the avoiding quadrant, 39.5% for the sensitivity quadrant, and 31.9% for the seeking quadrant.

### Differences Across the Sensory Modality Scores

With regard to the sensory system, the children with ASD demonstrated elevated and extremely elevated scores across five of the six sensory sections when compared with the normal range. As shown in Table 2, the children with ASD recorded higher scores for the auditory, touch, movement, body position, and oral sensory processing sections, which suggests that they experience difficulty regarding the meaningful use of these types of sensory information. The highest percentages were found for touch, with 84.9% of the children with ASD recording scores that met the criteria for classification as “much more than others” (MM2) or “more than others” (M1), closely followed by the scores for body position (73.1%), movement (68.9%), auditory (67.2%), and oral (57.1%) sensory processing. The lowest percentage was found for the visual processing Sect. (32.8%), which fell within the normal range.

### Differences Across the Behavioral Scores

In terms of the behavioral sections associated with sensory processing, significant differences were observed in three behavioral scores (conduct, social/emotional, and attentional responses) when comparing the children with ASD to norm-based expectations. This indicates that difficulties in the children’s behavioral responses might stem from sensory processing deficits.

Looking again at Table 2, 92.4% of the sample scored above the cut-off point for the social/emotional response section, closely followed by the attentional response Sect. (88.2%) and conduct Sect. (74.8%).

### Group-Related Differences Based on the CSP-2 Scores

Table 3 presents the group-related differences in the sensory processing characteristics of the children with ASD and the TD children, as measured using the four quadrants and two CSP-2 sub-sections (sensory and behavioral sections). On average, the results of the independent samples t-test indicated that the children with ASD scored higher on the CSP-2 than the TD children, thereby revealing more atypical sensory processing. The two groups differed significantly in terms of all the scores for the CSP-2 (all p < 0.001), with the exception of the visual sensory processing subscale. The mean scores in the TD group were classified as being within the typical performance range for all the assessed subscales, while the mean scores in the ASD group were classified as being in the atypical range for 12 of the 13 domains. The effect size calculations suggested the large magnitudes of the observed differences (the Cohen’s d effect sizes ranged from 1.65–3.68). Furthermore, the observed effect sizes indicated that the differences between the two groups would be both noticeable and relevant to practice. Among the four sensory quadrants, the most pronounced differences between the two groups were identified in relation to the registration pattern, followed by the avoiding, sensitivity, and seeking patterns. With regard to the six sensory systems, the most pronounced difference between the two groups was found in relation to the touch processing subscale. Finally, for the three behavioral sections, the most pronounced difference between the two groups was found for the social/emotional subscale.

**Table 3:** Comparing the ASD and TD Groups Based on the CSP-2 Scales

Subscale	ASD Sample (N = 119)		TDC Group (N = 30)		t-statistic (p)	Bonferroni correction	d
	M	SD	M	SD			
Sensory Quadrants (Bonferroni correction: α = 0.05/4 = 0.013)							
Seeking/Seeker	2.94	0.64	1.55	0.52	11.00***	0.01	2.38
Avoiding/Avoider	2.97	0.42	1.78	0.40	14.11***	0.01	2.90
Sensitivity/Sensor	2.71	0.46	1.45	0.42	13.64***	0.01	2.86
Registration/Bystander	2.80	0.64	1.24	0.27	20.28***	0.01	3.17
Sensory Section (Bonferroni correction: α = 0.05/6 = 0.008)							
Auditory Processing	3.27	0.64	2.17	0.69	9.32***	0.01	1.65
Visual Processing	2.54	0.67	2.36	0.40	1.92	ns	0.33
Touch Processing	2.75	0.70	1.19	0.27	19.15***	0.01	2.94
Movement Processing	2.83	0.85	1.64	0.53	8.85***	0.01	1.68
Body Position Processing	2.48	0.79	1.00	0.34	15.55***	0.01	2.43
Oral Sensory Processing	2.67	0.77	1.57	0.54	9.18***	0.01	1.65
Behavioural Section (Bonferroni correction: α = 0.05/3 = 0.016)							
Conduct	3.03	0.74	1.38	0.44	15.60***	0.02	2.71
Social/Emotional	3.04	0.46	1.52	0.36	16.73***	0.02	3.68
Attentional	3.14	0.55	1.40	0.40	16.20***	0.02	3.61

*p < 0.05, **p < 0.01, ***p < 0.001. The results are reported in this table using the means for the subscales so as to reflect the sums of the subscale item scores divided by the number of items for each subscale. This approach is in line with that of Little et al. (2018). The items are scored using a five-point Likert scale. The maximum possible score for each mean is five

### Relationship Between Sensory Processing Disorders and Age

To examine the effects of age on sensory processing disorders, linear regression analyses were performed to investigate the relationship between age and the discrepancy score for each of the nine CSP-2 scale raw scores. The results of the regressions are presented in Table 4.

**Table 4:** Linear Regression for Predicting the CSP-2 Scores by Age for Children with ASD

Subscale	Β	t	p	R2	AdR2	ƒ^2^	F
Sensory Quadrants							
Seeking	− 0.33	− 3.80	0.000^***^	0.110	0.10	0.12	14.465
Avoiding	− 0.29	− 3.24	0.002^**^	0.082	0.07	0.09	10.48
Sensitivity	− 0.38	− 4.40	0.000^***^	0.142	0.13	0.15	19.33
Registration	− 0.23	− 2.55	0.012	0.053	0.06	0.06	6.50
Sensory Section							
Auditory	− 0.39	− 4.53	0.000^***^	0.15	0.14	0.18	20.50
Visual	− 0.26	− 2.89	0.005^**^	0.07	0.06	0.07	8.33
Touch	− 0.17	− 1.87	0.064	0.03	0.02	0.03	3.50
Movement	− 0.45	− 5.37	0.000^***^	0.20	0.19	0.25	28.86
Body Position	− 0.26	− 2.92	0.004^**^	0.07	0.060	0.07	8.50
Oral	− 0.01	− 088	0.930	0.000	− 0.008	0.01	0.008
Behavioural Section							
Conduct	− 0.23	− 2.59	0.011	0.05	0.05	0.06	6.70
Social/Emotional	− 0.31	− 3.53	0.001^**^	0.096	0.09	0.11	12.45
Attentional	− 0.26	− 2.93-	0.004^**^	0.07	0.06	0.07	8.57

*p < .05, **p < .01, and ***p < .001

For the sensory quadrants, the linear regression revealed that age was a significant predictor of sensation seeking (β = -0.33, p < 0.001), sensation avoiding (β = -0.29, p < 0.002), and sensory sensitivity (β = -0.38, p < 0.001) behaviors, accounting for 10%, 7%, and 13% of the variance in these sensory patterns, respectively. This suggests that the sensory issues reduce as the children grow older. For the registration pattern, the standardized regression coefficient for age was greater than > 0.01, suggesting it to have hardly any effect.

Chronological age emerged as a significant predictor of some sensory systems, accounting for 14% and 19% of the variance in the auditory and movement domains for the children with ASD (F = 20.501, p < 0.001; F = 28.858, p < 0.001), which indicates moderate effect sizes. Although the regression coefficients for age and the visual and body position domains were statistically significant, age did not account for a sufficiently large percentage of the variance in those sensory processing aspects. Moreover, age-related effects were not found in relation to the touch and oral domains.

In terms of the behavioral section, similar results were found. There were statistically significant correlations between both the social and attentional domains and age, although the coefficient of age with the conduct domain was not statistically significant (p > 0.01).

Overall, the findings (six of the nine coefficients being statistically significant) indicate that as children with ASD age, the influence of their sensory symptoms decreases slightly.

## Discussion

### Prevalence of Sensory Abnormalities Among Children with ASD

This study examined the different sensory processing profiles seen in children with ASD. The findings indicate that children with ASD exhibit sensory differences in multiple sensory modalities, including auditory, touch, movement, body position, and oral sensory processing. These sensory processing differences demonstrate many patterns, including seeking, avoiding, sensitivity, and registration. Sensory differences in children with ASD may aggravate challenges associated with their behavioral, social/emotional, and attentional responses. The children with ASD in this study surpassed the clinical cut-offs for the normal ranges for all the CSP-2 sections, except for the visual sensory processing subscale. The children with ASD most commonly fell into the “more than others” and “much more than others” classifications, which indicate sensory processing difficulties. The sensory system scores with the highest identified differences were the touch, body position, movement, auditory, and oral scores, while the highest reported differences in the behavioral domains were seen in relation to the social/emotional, attentional, and conduct responses. Within the sensory quadrants, the highest reported differences were found for the registration, avoiding, sensitivity, and seeking quadrants. These findings regarding the children’s sensory symptoms are consistent with both expectations and prior findings, showing that children with ASD exhibit differences in their sensory processing abilities when compared with TD controls and other clinical groups (Ausderau et al., 2014; Cheung & Siu, 2009; Reynolds & Lane, 2008; Tomchek & Dunn, 2007; Tomchek et al., 2014). The findings are also consistent with those of previous studies confirming the prevalent and highly heterogeneous nature of the sensory domains in children with ASD (Ausderau et al., 2014; Ben-Sasson et al., 2009, 2019). Thus, the present study lends support for the inclusion of these sensory differences as a diagnostic criterion for ASD in the DSM-V, which states that people with ASD may experience “hyper- or hypo-activity to sensory input or unusual interests in sensory aspects of the environment” (APA, 2013a). Given the clinical controversy surrounding the diagnostic status of ASD, the results of this study indicate that the determination of a child’s sensory processing status should be a priority, as it may contribute to clinical decision-making.

One explanation for the prevalence of sensory symptoms in children with ASD relates to their hypo- or hypersensitivity to simulation, meaning that they do not react to sensory inputs in the same way as TD children (Baranek et al., 2006; Davis, 1990). Evidence suggests that children with ASD demonstrate significantly higher hyper- and hyposensitivities across all modalities when compared with TD children (Williams & Williams, 2011). Individuals who experience hypersensitivity have a low threshold for sensory stimuli, while individuals who experience hyposensitivity have a high threshold for such stimuli. Overall, the findings of this study indicate that sensory processing could be an indicator of developmental dysfunction in children with ASD.

### Sensory Quadrants

The sensory quadrant scores reflect patterns of low or high thresholds for the sensory inputs. As expected, the findings of this study show that the children with ASD more frequently engaged in the four sensory patterns: seeking, avoidance, sensitivity, and registration. The children with ASD tended to score in the “more than others” and “much more than others” ranges for all the sensory quadrants. These findings are consistent with those of prior studies indicating the sensory quadrants to be different in people with ASD when compared with TD controls (Kern et al., 2007; Leekam et al., 2007).

There is evidence that the atypical physiological arousal associated with ASD, which compromises the ability to regulate and exhibit an optimal response, may underlie the observed differences in the sensory quadrants, leaving people with ASD vulnerable to both hyper- and hyposensitivity to sensory stimuli in all the modalities (Ben-Sasson et al., 2008; Boucher, 2017; Orekhova & Stroganova, 2014; Schoen et al., 2009). Both types of responses can prove problematic for children with ASD, as functional participation in everyday life relies on a balance of activation so that an individual can be alert to stimuli that might prove a distraction from the task at hand (Dunn, 2014). When the brain is in a high state of arousal, it may have a low threshold for certain types of sensory stimuli. In other words, an atypical level of a certain sensation may lead to an overly strong reaction in the brain (Griffin, 2013). Thus, children with ASD may exhibit an amplified active or passive response to sensory inputs and, therefore, be “classed as avoiders or sensors,” respectively (Whitman, 2004).

By contrast, when the brain is in a low state of arousal, it may have a much higher threshold for certain sensory stimuli. As a consequence, children with ASD tend to respond passively to sensory inputs or even fail to notice normal sensory stimuli that easily evoke responses in others, which results in children with ASD being classed as bystanders (Dunn, 2007). Conversely, children with ASD may respond actively to sensory stimuli in order to stimulate their sensory system and so be “classed as seekers.” (Dunn, 2014).

When considering individual ranges beyond what is classed as normal, the findings of this study suggest that the most common atypical sensory patterns in the “much more than others” range were associated with problems related to registration. The second most significant area of difficulty in this regard was the avoiding quadrant, followed by the sensitivity and seeking quadrants. These findings appear to support the notion that the registration pattern represents the most common pattern of atypical sensory-related behavioral problems reported in children with ASD (Ausderau et al., 2016; Baranek et al., 2006, 2014; Ben-Sasson et al., 2007; Rogers & Ozonoff, 2005; Schoen et al., 2009). For instance, in a meta-analysis of 14 studies, Ben-Sasson et al. (2009) concluded that a significant difference existed between the ASD and TD groups in terms of the presence/frequency of the observed sensory symptoms, with the greatest difference being seen in relation to under-responsivity, followed by over-responsivity and sensation seeking. By contrast, other studies have reported that hyposensitivity does not discriminate between children with ASD and children with other developmental disorders (Cheung & Siu, 2009; Ermer & Dunn, 1998; Little et al., 2018; Rogers et al., 2003).

One possible neurological explanation for the under-responsiveness to sensory stimuli seen in individuals with ASD is the depression of sensory afferents in the cerebellum, which results in inconsistent sensory modulation (Kern, 2002). Little et al. (2018) suggested that the difficulty in registration seen in children with ASD may be related to altered executive functioning and attentional mechanisms. This suggests a lack of “top-down” cognitive control of the attentional mechanisms implicated in orienting to relevant sensory stimuli.

However, this study found the avoiding pattern to be the most common problem (94.1%) seen in relation to atypical sensory patterns when considering both the “much more than others” and “more than others” ranges. This finding is in line with the notion that hypersensitivity represents the most impaired response in those with ASD (O’Brien et al., 2009). Relatedly, in a recent meta-analysis of 55 questionnaire studies concerning the sensory symptoms observed in those with ASD, Ben-Sasson et al. (2019) determined the most consistent sensory experience in individuals with ASD to be hypersensitivity. It has been posited that this hypersensitivity to sensory stimuli is due to the enhancement of sensory afferents in the cerebellum, which results in inconsistency in the intensity or modulation of sensory information.

Although several prior studies have reported a higher frequency of sensory seeking and sensory hypersensitivity to be common among the ASD population (Baranek et al., 2006; Dunn et al., 2002; Jasmin et al., 2009; Nieto et al., 2017), such sensory problems are not limited to ASD, as they are also present in other developmental disorders (Baranek et al., 2006). In addition, related problems have been attributed to a lower developmental level and younger chronological age, rather than being specific to ASD (Rogers & Ozonoff, 2005).

This conflict regarding sensory processing patterns may be more distinctive in those with ASD, and it might result from the age variable. Some studies have suggested that age impacts atypical sensory profile processing patterns (Ben-Sasson et al., 2009, 2019), although other studies have not found support this hypothesis (McCormick et al., 2016). According to Nieto et al. (2017), an alternative explanation for the difference in prior results concerning the atypical sensory subtypes that may be specific to ASD is the sensory environment at both the micro-level (family routines) and the macro-level (cultural environment). In other words, it might be related to how these sensory features relate to the interplay between the nature of the specific difficulties faced and the context in which a child is raised.

It may prove difficult to identify any specific sensory patterns as being characteristic of ASD. Children with ASD tend to display mixed types of sensory responses, and they frequently engage in all four sensory quadrants (Ashburner et al., 2008; Baranek et al., 2006; Ben-Sasson et al., 2007; Cheung & Siu, 2009; Tomchek & Dunn, 2007). Thus, it should be recognized that it is the frequency of sensory symptoms rather than their systematic form that distinguishes individuals with ASD from both TD individuals and other clinical groups (Uljarević, 2013).

### Sensory System Section

The present results reveal touch processing to be the most severely affected sensory modality in children with ASD, followed by the body position, movement, auditory, and oral modalities. This is in line with the findings of prior studies indicating problems with touch stimuli to be the most prevalent sensory problems in individuals with ASD (Chang et al., 2014; Foss-Feig et al., 2012; Puts et al., 2014; Rogers et al., 2003; Tomchek & Dunn, 2007). One potential explanation for the difference in tactile processing seen in children with ASD concerns the altered γ-aminobutyric acid function associated with ASD (Puts et al., 2017). Indeed, a growing body of evidence indicates that this neurotransmitter plays an important role in the neuronal response to tactile stimulation (Mikkelsen et al., 2018). However, evidence also exists that no abnormalities regarding tactile processing are found in children with ASD (Cascio et al., 2008; Güçlü et al., 2007; O’Riordan & Passetti, 2006). This discrepancy may stem from differences in study protocols, for example, while this study relied on proxy reports, studies that failed to identify differences in participants’ touch responses relied on an experimental design.

The results of this study also suggest the body position modality to be associated with the second highest prevalence in terms of the sensory problems experienced by children with ASD, closely followed by the movement modality. This finding is somewhat surprising, as some prior studies have indicated the body position and movement modalities (Ricon et al., 2017; Tomchek & Dunn, 2007) to be much less regularly subject to dysfunction in individuals with ASD. Yet, it is consistent with the results obtained by Al-Heizan et al. (2015), which indicated that more than half of the tested children with ASD demonstrated difficulties with regard to the body position and movement domains. While possible explanations for the relatively high frequency of sensory problems seen in relation to these two sensory domains are not yet well elaborated, in the case of the present study it seems feasible that the explanation might be related to the culture and community involved (Al-Heizan et al., 2015). The parenting style most commonly practiced in the Arab Gulf region involves the overprotection and careful nurturing of children, which could be a causative factor with regard to limiting children’s opportunities to be exposed to physical stimuli (Al-Heizan et al., 2015). Alternatively, it is possible that parents in the Gulf region might be more sensitive to dissocial behavior and, therefore, tend to overestimate it (Amr et al., 2012).

More than two-thirds of participants (67.2%) in this study were classified as atypical in terms of their auditory processing. This is consistent with the results of prior studies revealing that children with ASD exhibit auditory processing abnormalities (Chang et al., 2014; Kern et al., 2006; Little et al., 2018; O’Connor, 2012; Tomchek & Dunn, 2007). Often, these abnormalities manifest in children with ASD as an adverse response to an unexpected stimulus. The ability to acquire and parse a variety of incoming sounds forms the foundation for both language and communication (Marco et al., 2011). When auditory processing is performed less efficiently in the brain or when sounds are blocked out, it can affect language development and contribute to the kinds of communicative difficulties seen in children with ASD (Linke et al., 2018; Marco et al., 2011; O’Connor, 2012).

It has previously been suggested that the auditory processing abnormalities exhibited by children with ASD may stem from the abnormal cortical processing of auditory information (Ross-Swain, 2018). In fact, functional magnetic resonance imaging studies have shown that the auditory processing difficulties experienced by children with ASD may be associated with a reduction in the interhemispheric functional connectivity of the auditory cortices (Linke et al., 2018). Gomot et al. (2008) noted the increased activation of the right prefrontal/premotor and left inferior parietal regions in children with ASD during target detection. Moreover, it has been suggested that the abnormal processing of auditory stimuli exhibited by individuals with ASD might reflect the immaturity of their central auditory nervous system (Kwon et al., 2007). In addition, Boddaert et al. (2004) reported that the auditory deficits seen in children with ASD may be due to the reduced activation of different areas of the left hemisphere of the brain, including the speech-related areas.

The results of this study also suggest that more than half of the children with ASD experienced abnormal oral sensory processing. Such problems can manifest in different ways, including the avoidance of certain foods and an aversion to oral activities such as tooth brushing. The present findings regarding oral sensory sensitivity are consistent with those of other studies demonstrating abnormal oral sensory responses in children with ASD (Chistol et al., 2018; Hazen et al., 2014; Nadon et al., 2011; Tomchek & Dunn, 2007). These kinds of oral processing problems often accompany feeding disorders in children with ASD (Chistol et al., 2018; Kral et al., 2015; Smith, 2016). Indeed, there is evidence that the prevalence of feeding difficulties in children with ASD may range from 60%–90% (Padmanabhan & Shroff, 2018). One possible explanation for these oral problems concerns the limited opportunities available for oral exploration during infancy (Bern & Minando, 2016), when the mouth is considered the primary tool for reception stimulation and pleasure (Hockenberry & Wilson, 2014). The continuous rejection of foods with specific textures can prevent the acquisition of developmentally appropriate oral motor skills, especially if the oral sensory deprivation occurs during the period in which an infant progresses from reflexive to learned and voluntary feeding actions (Bern & Minando, 2016; Hockenberry & Wilson, 2014). Moreover, conditions associated with gastrointestinal discomfort, as well as negative associations formed through adverse oral or feeding experiences, have also been suggested as possible explanations for oral sensory problems (Bern & Minando, 2016). Interestingly, the present results contrast with those of Shah et al. (2015), who found that oral sensory sensitivity was not among the most common sensory issues experienced by children with ASD. The authors explained their results with reference to cultural differences surrounding food, which could contribute to reducing the likelihood of oral sensory dysfunction (Shah et al., 2015).

In terms of the visual domain, the children with ASD in this study recorded visual processing scores within the normal range when compared with the normative data. This is consistent with prior findings indicating visual processing to be an area of relative strength for most children with ASD (Dellapiazza et al., 2019; Little et al., 2018). However, this finding regarding the typical visual modality observed in children with ASD contrasts with the results of some prior studies that used other measures and identified the presence of atypical visual processing in individuals with ASD (Leekam et al., 2007). Such atypical visual behaviors manifest in children with ASD as the avoidance of visual input or the seeking out of visual stimuli (Marco et al., 2011). According to Dellapiazza et al. (2019), one possible explanation for the absence of visual processing symptoms in children with ASD relates to the fact that the atypical visual behaviors considered by the CSP-2 are difficult for parents to assess.

### Sensory Behavioral Section

The results of this study show that the children with ASD scored outside the normal range in relation to those behaviors associated with sensory processing. This finding is consistent with the results of prior studies that identified differences in the behaviors associated with sensory processing symptoms between children with and without ASD (Little et al., 2018). Thus, it appears that inaccurate sensory processing can adversely affect the behavioral, attentional, and social-emotional responses of children with ASD.

In fact, the results of this study suggest that a large majority of the sample (92%) experienced difficulties with their emotional/social responses. In general, children with ASD are characterized by marked deficits in their social communication behavior, although the CSP-2 items do not measure broader social deficits. Two explanations have been proposed for these findings concerning social outcomes. First, hyporesponsiveness in the social context may be associated with distractibility (Brock et al., 2012). For example, children with ASD tend to engage in more sensory experiences, which leads to sensory distraction in the socio-communicative environment, thereby resulting in them missing social cues and finding social situations more confusing and stressful (Gaines et al., 2016). Second, hyperresponsiveness in the social context may be related to increased withdrawal (Brock et al., 2012). For example, children who engage in less sensory experiences may withdraw from over-stimulating socio-communicative environments, leading to them having less practice with social scenarios and failing to successfully engage in social-communicative interactions (Thye et al., 2017).

The results also indicate that most of the children with ASD (88%) experienced difficulties with regard to their attentional responses. It has been reported that the atypical sensory processing patterns seen in children with ASD exhibit interrelations with attentional difficulties (Dellapiazza et al., 2018). In fact, atypical sensory processing has been found to be associated with disturbances in attention disengagement (Sabatos-DeVito et al., 2016). For example, children who exhibit the registration pattern may miss or be slow to identify sensory stimuli that appear outside their area of attention. Thus, they may require support to notice important stimuli and react appropriately to them. Moreover, children with ASD who engage in sensory-seeking behaviors may be easily distracted by other stimuli. However, sensory hyperresponsiveness can co-occur with overfocused attention (Liss et al., 2006). For example, children with ASD who exhibit hyperresponsiveness tend to avoid overfocusing on a given stimulus by engaging in ritualistic patterns so as to avoid sensory overload, which leads to the failure to focus attention and recognize naturally occurring and important stimuli.

Additionally, the findings of this study indicate that nearly three-quarters (74.8%) of the sample exhibited difficulties in terms of their conduct outcomes, which suggests that challenges associated with sensory processing––both hyperresponsiveness and hyporesponsiveness––can affect a child’s behavioral responses in everyday life and be misinterpreted as maladaptive behaviors. This finding is supported by the results of previous studies concerning the impact of atypical sensory processing on behavioral problems (Dellapiazza et al., 2018; Suarez, 2012; Tomchek et al., 2015). It has been observed that children with ASD who exhibit sensory processing difficulties tend to experience problems completing tasks or producing quality work. One possible explanation for this finding concerns the fact that sensory processing dysfunctions can interfere with a child’s ability to perform or participate in the age-appropriate activities of daily life, as such activities require spontaneous and appropriate sensory interaction.

### Relationship Between Age and Sensory Processing Characteristics

This study also sought to determine whether the observed sensory processing abnormalities changed as the children with ASD aged. The results indicate that the participants’ age was correlated with the sensory patterns (seeking, avoiding, and sensitivity), sensory systems, and behavioral scores, except for the registration, touch, oral, and conduct processing subscales. These statistically significant correlations suggest that older children with ASD exhibit fewer difficulties in relation to their responses to sensory stimuli. Thus, the data offer evidence that the developmental level varies with age. This finding is consistent with the general decreasing pattern of abnormal sensory processing with age observed in the ASD population (Kern et al., 2006) and the partial correlations found by Ben-Sasson et al. (2009) between some sensory patterns (hypersensitivity and seeking) and age. Yet, these results contrast with those of studies suggesting that the unusual sensory processing characteristics seen in children with ASD do not dissipate with age (Billstedt et al., 2007; Crane et al., 2009; Perez Repetto et al., 2017).

One possible explanation for the age-related differences identified in the present study is the mirroring of the typical development of sensory responses, which likely results in a reduction in sensory symptoms as the development and use of functional behaviors occur. It is feasible that many of these sensory responses lessen in some children with ASD as they age due to the maturation process that occurs over time and at an individual rate. However, the accuracy of this potential explanation needs to be confirmed by means of longitudinal analyses of sensory symptoms (Kay, 2001; Kern et al., 2007).

Alternatively, the age-related differences could be related to the individual compensation and coping mechanisms that children with ASD develop as they age so as to decrease the impact of unpleasant or overwhelming sensory experiences (Little et al., 2018). Moreover, environmental modifications made by parents, teachers, or service providers could also contribute to the decrease in children’s abnormal responses to sensations (Little et al., 2018).

## Conclusion

Although the current findings must be regarded as tentative given the sample size, this study potentially provides additional evidence concerning both the prevalence of sensory processing disorders among children with ASD and the broad nature of such disorders. The findings appear to confirm the multimodal nature of the sensory disturbances seen in children with ASD. Such disturbances are likely to appear across different sensory modalities, including the auditory, touch, movement, body position, and oral sensory processing modalities. Furthermore, they can take many forms, such as registration, sensitivity, avoiding, and seeking. The findings of this study also indicate that the impacts of sensory disturbances and the associated difficulties likely extend to other behavioral areas, including conduct, social/emotional, and attentional behaviors. In addition, the present findings suggest that age may represent a confounding variable when it comes to the assessment of sensory processing characteristics. Indeed, the findings seem to indicate correlation between sensory processing issues and age, thereby demonstrating that sensory symptoms may differ with chronological age. Thus, this study provides some initial support for the hypothesis that sensory symptoms may decrease over time.

Moreover, it tends to confirm and extend the findings of a prior study regarding the prevalence and types of sensory processing disorders seen among a relatively large sample of non-Western individuals with ASD (Al-Heizan et al., 2015).One strength of this study relates to the decision to focus on the different sensory patterns and modalities, rather than to adopt the traditional focus on a single sense or limited subset of sensory symptoms, as well as the decision to focus on the behaviors associated with different sensory processing scores. This approach allowed for the collection of richer data and the performance of a more in-depth analysis. That said, this study did have several limitations, including the fact that the assessment of sensory processing was limited to caregiver ratings. Although such subjective measurements can provide valuable information regarding sensory processing difficulties in everyday contexts, they are not considered the ideal assessment measure. The kind of frequency questionnaires used in this study may be subject to response bias, meaning that future studies should include objective measures of sensory processing in addition to informant reports so as to allow for a complementary evaluation. The inclusion of multi-informant ratings of sensory symptoms in future studies may help to further elucidate the exact nature of the sensory processing disorders seen in individuals with ASD. Obtaining information from multiple sources and different informants represents a more preferable means of reflecting different perspectives regarding the various aspects of children’s sensory processing. It also allows for different perspectives to be collected within a more ecologically valid framework. The results of this study concerning the impact of age on sensory processing disorders must be considered tentatively, as the data reflect the assessment of sensory aspects at a single point in time. Longitudinal studies would help to extend understanding of the developmental perspective on sensory processing in children with ASD and the impact of such disruptions. In terms of the sample, the present study only included children diagnosed with mild or high-functioning ASD. As a consequence, the findings may not be generalizable to the broader ASD population. To confirm and extend the findings of this work, future studies should employ samples that include a broader range of ASD severity.

Despite the above-mentioned limitations, the findings of this study have valuable implications for both the theoretical and clinical contexts. The findings indicate that sensory symptoms are among the earliest risk markers to emerge in children later diagnosed with ASD (Robertson & Baron-Cohen, 2017). The effects of such symptoms are also likely to extend to other developmental areas. Thus, sensory symptoms and markers should be included in the general evaluation protocols related to ASD. In fact, it is important to recognize that sensory processing differences have clinical value and can guide clinicians in making more informed judgements regarding onward referral and diagnosis.

Moreover, it should prove beneficial for both parents and professionals who work with children with ASD to recognize the heterogeneity that exists in terms of sensory processing among the ASD population and, therefore, to avoid a one-size-fits-all intervention approach. Such recognition should help to further classify the symptom clusters into relatively smaller, more homogeneous, and more meaningful subgroups and so to foster an optimum approach to service delivery. Furthermore, increasing parents’ awareness of their child’s limitations and strengths in relation to sensory processing could contribute to better directing interventions toward practical aspects that are appropriate in real-life contexts.
