# Correction: Caregivers' Assessment of the Sensory Processing Patterns Exhibited by Children with Autism in the Gulf Region

**DOI:** 10.1007/s10803-024-06679-7

**Published:** 2024-12-20

**Authors:** Rehab H. Alsaedi, Suzanne Carrington, James J. Watters

**Affiliations:** 1 Faculty of Creative Industries, Education and Social Justice, Queensland University of Technology (QUT), Victoria Park Road, Kelvin Grove, Brisbane, QLD 4059 Australia; 2 The Department of Special Education, Taibah University, Janadah Bin Umayyah Road, Madinah, 41477 Saudi Arabia

**Correction to: J Autism Dev Disord** 10.1007/s10803-023-05937-4

Please note the information that was inadvertently omitted from the article regarding Acknowledgments:

**Acknowledgments**

The authors would like to thank the parents and staff members from the participating autism centers and mainstream primary schools in Bahrain, Saudi Arabia, and the UAE for their assistance with the data collection. This study was conducted as part of the first author’s Ph.D. thesis. The first author also gratefully acknowledges the original supervisory team, Professor Karen Sullivan and Professor Linda Gilmore, for their guidance during the initial stages of the Ph.D. candidature project.

